# The Effects of an Online Mindfulness Intervention on Perceived Stress, Depression and Anxiety in a Non-clinical Sample: A Randomised Waitlist Control Trial

**DOI:** 10.1007/s12671-018-0925-0

**Published:** 2018-05-02

**Authors:** Dawn Querstret, Mark Cropley, Chris Fife-Schaw

**Affiliations:** School of Psychology, University of Surrey, Guildford, Surrey, GU2 7XH UK

**Keywords:** Mindfulness, Online intervention, Stress, Depression, Anxiety

## Abstract

Mindfulness interventions have been shown to be effective for health and wellbeing, and delivering mindfulness programmes online may increase accessibility and reduce waiting times and associated costs; however, research assessing the effectiveness of online interventions is lacking. We sought to: (1) assess the effects of an online mindfulness intervention on perceived stress, depression and anxiety; (2) assess different facets of mindfulness (i.e. acting with awareness, describing, non-judging and non-reacting) as mechanisms of change and (3) assess whether the effect of the intervention was maintained over time. The sample was comprised of 118 adults (female, *n* = 95) drawn from the general population. Using a randomised waitlist control design, participants were randomised to either an intervention (INT) or waitlist control (WLC) group. Participants completed the online intervention, with the WLC group starting after a 6-week waitlist period. Participants completed measures of depression (PHQ-9), anxiety (GAD-7) and perceived stress (PSS-10) at baseline, post-treatment, 3- and 6-month follow-up. Participants who completed the mindfulness intervention (*n* = 60) reported significantly lower levels of perceived stress (*d* = − 1.25 [− 1.64, − 0.85]), anxiety (*d* = − 1.09 [− 1.47, − 0.98]) and depression (*d* = − 1.06 [− 1.44, − 0.67]), when compared with waitlist control participants (*n* = 58), and these effects were maintained at follow-up. The effect of the intervention was primarily explained by increased levels of non-judging. This study provides support for online mindfulness interventions and furthers our understanding with regards to how mindfulness interventions exert their positive effects.

## Introduction

There is a growing evidence-base for the efficacy of mindfulness courses—be that Mindfulness Based Stress Reduction (MBSR; Kabat Zinn 1982) or Mindfulness Based Cognitive Therapy (MBCT; Segal et al. 2002)—for a range of chronic health problems such as depression, chronic pain and anxiety disorders (Grossman et al. 2004). Research has also shown that perceived stress decreases after taking part in a mindfulness intervention with benefits maintained at follow-up between 1 and 3 months (Carmody et al. 2009; Krusche et al. 2012). However, with the exception of two studies which assessed online interventions (Gluck and Maercker 2011; Krusche et al. 2012), previous studies considering the effect of mindfulness on perceived stress have assessed group-based face-to-face mindfulness interventions. As awareness of the benefits of mindfulness-based therapy increases, so does the need to improve access to these types of interventions. One way to increase access is to deliver interventions online. In addition to reducing the costs and decreasing waitlist times, operationalising interventions online enables participants to complete them from the comfort of their own home and in their own time. Furthermore, accessibility could be improved for a large number of people who may benefit but who may not be able to physically attend a face-to-face course (Finucane and Mercer 2006).

However, there are challenges associated with proposing the delivery of mindfulness courses online. Traditionally, mindfulness interventions are conducted face-to-face (in groups) facilitated by mindfulness trainers. Developers of mindfulness-based interventions suggest that the presence of others is an important part of the learning process because, not only do other group members provide social support, they also learn from engaging in investigative dialogue (between the teacher and group members) at the end of each class (Kabat-Zinn 1990; Segal et al. 2002). Indeed, this position is supported by qualitative studies suggesting that participants find the group context largely very helpful (e.g. Allen et al. 2009; Mason and Hargreaves 2001).

While in theory online mindfulness interventions may be a cost-effective way forward, there is limited research assessing their efficacy. In a pilot study, Gluck and Maercker (2011), employing a randomised waitlist control design, assessed the effect of a 2-week web-based mindfulness intervention on perceived stress, distress and mindfulness and reported that the intervention showed a non-significant trend of improvement for all measures (with medium effect sizes; *d* = 0.46–0.77). Krusche et al. (2012), in a pre-post study, assessed an online mindfulness course for its effect on perceived stress and reported a significant reduction with a large effect size (*d* = 1.57) which was stable at 1-month follow-up. The lack of a control group does raise the question as to whether or not this was a general treatment effect; however, the effect size was of similar magnitude to other studies assessing face-to-face mindfulness interventions for stress. For example, Shapiro et al. (2007), employing a randomised waitlist control design, assessed the impact of an MBSR intervention, found a medium between-groups effect size (*d* = − 0.70), and in another randomised waitlist control trial, the authors reported a large between-group effect size for an MBSR intervention (*d* = 0.91, Nyklicek and Kuijpers 2008). Furthermore, a recent meta-analysis focussing exclusively on studies assessing online mindfulness-based interventions (*n* = 15; predominantly MBSR), reported small but beneficial effects on depression (*g* = 0.29), and anxiety (*g* = 0.22), and moderate effects for the reduction of stress (*g* = 0.51; Spijkerman et al. 2016).

Understanding how mindfulness works will help us to identify those for whom it will be most effective. However, few studies are designed to understand “why” and/or “how” mindfulness delivers its benefits (Glomb et al. 2011); therefore, intervention studies need to be constructed in order to assess possible mechanisms of change. Shapiro et al. (2006) suggest two approaches. Firstly, dismantle (tease apart) studies can separate out and compare various active ingredients in mindfulness-based interventions, and secondly, studies can examine the central construct of mindfulness to establish whether the development of “mindfulness” (or different facets of mindfulness) leads to the positive changes that have been observed. This step can be facilitated by employing valid and reliable measures of mindfulness for use in statistical models of mediation.

Rounsaville and Carroll’s (2001) three-stage model of behavioural therapies research articulates progressive stages of development and evaluation of behavioural treatments. Stage one focusses on the development of the intervention and conducting feasibility and pilot trials; Stage two focusses on conducting initial efficacy trials to evaluate manualised and pilot-tested treatments which have shown promise; and Stage three focusses on conducting larger RCTS with well-chosen control groups to establish generalisability, implementation challenges and cost-effectiveness. Similarly, in the National Institutes Health Stage Model (Onken et al. 2014), a six-stage progressive model is proposed in the development of behavioural therapies. The current study is situated in stage 2 according to Rounsaville and Carroll, and to the equivalent stage (stage three) in the National Institutes Health Stage Model. We predicted that, in comparison to participants in the waitlist control group, participants who completed the mindfulness course would report significantly lower levels of perceived stress (H1), depression (H2) and anxiety (H3), immediately after course completion. We also sought to explore whether any treatment gains were maintained at 3 and 6-month follow-up (H4).

## Method

### Participants

The sample was comprised of 118 participants (intervention group, 60; waitlist control group, 58) recruited from the general population. With an age range of 21–62 years (*M* = 40.68, SD = 10.45), 95 participants (80.5%) were female. The majority of participants (94.9%; *n* = 112) worked full-time for a mean of 45.12 (SD = 14.84) hours/week and were married or had a partner (*n* = 85; 72%), with 59 (50%) having dependent children. One hundred and two participants (86.4%) worked a 9 am–5 pm (Mon-Fri) pattern, with the remaining participants working shifts. Many job roles were represented including: nursing/medicine (26.3%; *n* = 31), healthcare (e.g. dieticians, physiotherapists; 20.3%; *n* = 24), administration (19.5%; *n* = 23), education (e.g. teachers, University lecturers; 14.4%; *n* = 17), management (8.5%; *n* = 10), police (6.8%; *n* = 8) and other (4.2%; *n* = 5). Roughly two thirds of participants were University educated (68.6%; *n* = 81).

**Fig. 1:**
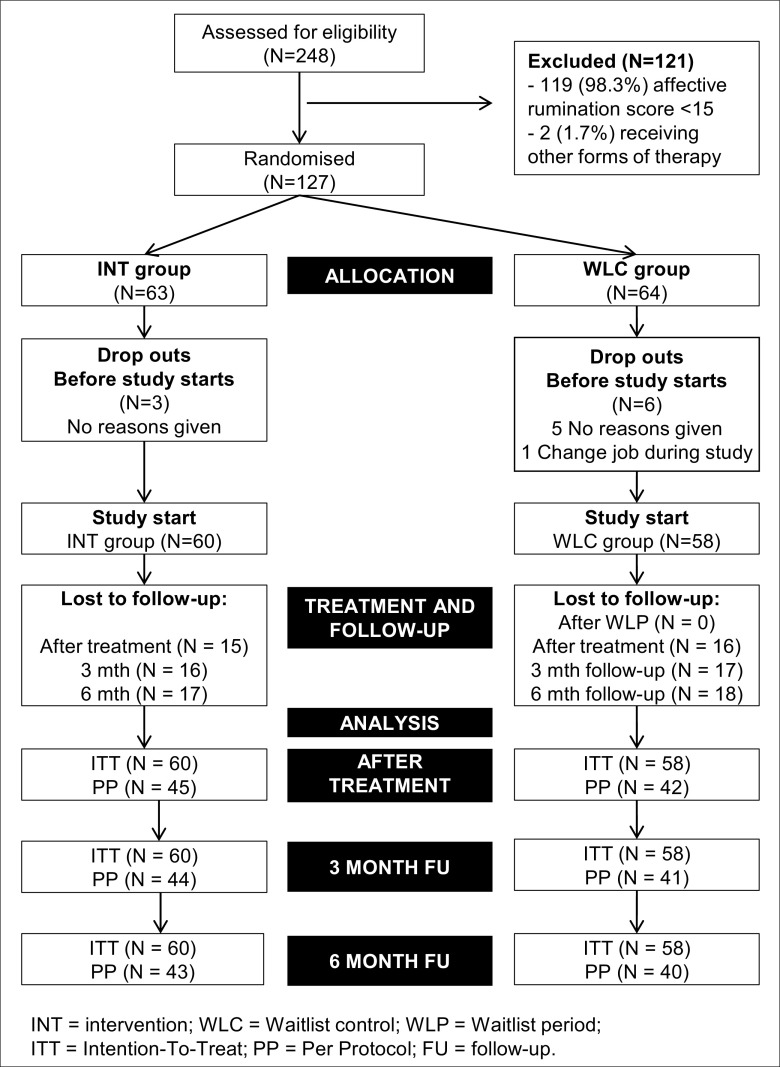
CONSORT flow diagram

Approximately 50% of the sample reported moderate to severe levels of depression and/or anxiety symptoms at baseline (see Table 1); however, only five participants self-identified as having depression or anxiety (three from the intervention group, and two from the waitlist control group), and two of these participants stated they were taking medication. Participants attracted to taking part in this study may have been seeking help due to the severity of experienced symptoms. As part of the consenting process, participants agreed to complete the course within 4 weeks if possible. See Fig. 1 for the participant flow from screening to follow-up, and Table 1 for sample specifics for each of the study groups.

**Table 1:** Demographic and clinical characteristics for intervention and waitlist control groups at baseline

Variable	Group		Group difference		
	INT (*n* = 60)	WLC (*n* = 58)	*t*	*X* ^2^	*p*
Total number females (%)	48 (80%)	47 (81%)		0.02	0.89
Age range in years (M; SD)	21–62 (41.67; 10.57)	21–60 (39.66; 10.33)	− 1.04		0.29
Number working full-time (%)	55 (91.7%)	57 (98.3%)		2.66	0.11
Mean hours per week (SD)	42.12 (12.84)	44.04 (13.81)	0.88		0.11
Number married/living with partner (%)	41 (68.4%)	38 (65.5%)		0.83	0.36
Number with children (%)	34 (56.7%)	25 (43.1%)		2.17	0.14
Number university educated (%)	44 (73.3%)	37 (63.8%)		1.25	0.26
Moderate/severe depression	34 (56.6%)	27 (46.5%)		1.21	0.27
Moderate/severe anxiety	29 (48.3%)	24 (41.4%)		0.57	0.46

Job types (*N* [%]): INT group—nursing/medicine (11 [18.3%]), healthcare (14 [23.3%]), administration (13 [21.7%]), education (11 [18.3%]), management (3 [5.0%]), police (5 [8.3%]), psychology (2 [3.3%]), other (1 [1.7%]); WLC group—nursing/medicine (15 [25.9%]), healthcare (10 [17.2%]), administration (10 [17.2%]), education (6 [10.3%]), management (7 [12.1%]), police (3 [5.2%]),psychology (4 [6.9%]), other (3 [5.2%])

*INT* intervention group, *WLC* waitlist control group

### Procedure

#### Experimental Design

A randomised waitlist control design was employed. Participants were assessed pre-treatment and post-treatment and were followed up at 3 and 6 months post-treatment.

#### Recruitment

Details of the study were circulated to organisations within the UK with which the University had relationship in order to promote the study to their staff. For reasons of confidentiality, the specific organisations cannot be named but they span the following industry sectors: Healthcare (e.g. nursing, medicine), Policing, Legal, Education, Information Technology and Telecommunications. In addition, the study was promoted via social media and was also advertised on an online professional networking site (www.LinkedIn.co.uk).

#### Screening

Individuals completed an online screening questionnaire. To be eligible for inclusion, participants had to (1) be 18 years of age or older; (2) be working a minimum of 30 h per week; (3) have the ability to commit to 2 h (minimum) per week for the duration of the course; (4) have access to the Internet at home; (5) not be receiving any other form of psychological therapy and no plans to start any other form of therapy throughout the duration of the study; (6) have no previous experience of mindfulness or meditation; (7) agree to maintain any dosage of existing medication during the study, but in the event that dosage needs to change during the study for medical reasons to notify study personnel; and (8) be living and working in the UK.

#### Randomisation Process

Randomisation did not occur until all participants had registered for the study. Allocation concealment was achieved by allocating unique identifiers to each participant and then randomly sorting the file in SPSS version 21 (IBM Corp 2012). Participants, using the unique identifiers, were then randomly assigned into blocks of four (stratified by gender) which were generated using a random number generator program (Urbaniak and Plous 2013). Allocation to even numbers in the block denoted intervention group membership, and to odd numbers denoted waitlist control group membership. We stratified by gender because previous research has highlighted gender differences regarding the prevalence and severity of anxiety and depression (McLean et al. 2011). Participants were blinded to group membership. They were not able to choose which group they were allocated to however they were informed there were two course start dates. Participants had no contact with each other because all recruitment was conducted online, and all communication with participants was conducted via personal email. The data set used for analysis contained only an anonymised participant unique identifier for each participant.

#### Compensation for Participation

Participants were offered £50 worth of Love2Shop vouchers to compensate them for their time and for expenses associated with completing the course. In addition, participants were informed that they were completing an online course for free which would normally cost them £30.

#### Online Mindfulness Course

The online mindfulness-based cognitive therapy (MBCT; Teasdale et al., 2000) course (www.bemindfulonline.com) is run by the Mental Health Foundation (UK) and Wellmind Media (UK), and was developed in conjunction with leading UK mindfulness instructors (Krusche et al. 2012). The course usually costs £30 per person; however, participants in this study were able to complete the course for free. The course is modelled on the class sequence of traditional MBCT programmes. In the current study, participants were asked to complete the course within 4 weeks if possible. Participant completion was tracked throughout the course. Participants were sent reminder emails when they had not accessed the course for more than a week. Participants did not have any personal contact with the mindfulness instructors at any point during the course. All instructional video and audio files were embedded within the website. For further detail regarding the course, see Krusche et al. (2012) and Querstret et al. (2016).

### Measures

#### Perceived Stress

The Perceived Stress Scale 10 item (PSS-10; Cohen et al. 1983) consists of 10 items (e.g. “In the last month, how often have you felt that you were unable to control the important things in your life?”) which are answered using a five-point Likert scale, ranging from 0 (Never) to 4 (Very often). A total score is computed by summing the scores on the individual items with scores ranging from 0 to 40. Cronbach’s alphas: T1 = 0.86, T2 = 0.88, T3 = 0.91, T4 = 0.92.

#### Depression

The Patient Health Questionnaire 9-item (PHQ-9; Kroenke et al. 2001). Participants are asked to consider over the last 2 weeks, how often they have been bothered by a list of nine problems, for example, “Little interest or pleasure in doing things”. Items are scored against a Likert scale ranging from 0 (Not at all) to 3 (Nearly every day) with summed today scores ranging from 0 to 27. Depression severity is determined on the basis of the total score, as follows, 0–4 = No depression; 5–9 = Mild depression; 10–14 = Moderate depression; 15–19 = Moderately severe depression; and 20–27 = Severe depression. Cronbach’s alphas: T1 = 0.88, T2 = 0.89, T3 = 0.89, T4 = 0.87.

#### Anxiety

The Generalised Anxiety Disorder 7-item (GAD-7; Spitzer et al. 2006). Participants are asked to consider over the last 2 weeks, how often they have been bothered by a list of seven problems, for example, “Feeling nervous, anxious, or on edge”. Items are scored against a Likert scale ranging from 0 (Not at all) to 3 (Nearly every day) with summed total scores ranging from 0 to 21. Anxiety severity is determined on the basis of the total score, as follows, 0–4 = No anxiety; 5–9 = Mild anxiety; 10–14 = Moderate anxiety; and 15–21 = Severe anxiety. Cronbach’s alphas: T1 = 0.92, T2 = 0.92, T3 = 0.92, T4 = 0.90.

#### Mindfulness

The Five Facet Mindfulness Questionnaire Short form (FFMQ-SF; Bohlmeijer et al. 2011) has 24-items that measure five facets of mindfulness: *observing* (OBS; 4 items, e.g. I notice the smells and aromas of things), *describing* (DES; 5 items, e.g. I’m good at finding the words to describe my feelings), *acting with awareness* (AA; 5 items, e.g. It seems I am “running on automatic” without much awareness of what I am doing), *non-judging* (NJ; 5 items, e.g. I criticise myself for having irrational or inappropriate emotions) and *non-reactivity* (NR; 5 items, e.g. I watch my feelings without getting lost in them). Participants are asked to rate the degree to which each statement is true for them. Items were scored on a five-point Likert scale ranging from 1 (never or very rarely true) to 5 (often or always true), with summed facet scores ranging from 5 to 25. Previous research has shown that the OBS facet is only predictive for participants with previous experience of meditation (Baer et al. 2006), and the participants in the current study were required to be naive to meditation; therefore, this facet was not utilised. Cronbach’s alphas: DES (T1 = 0.84, T2 = 0.85); AA (T1 = 0.79, T2 = 0.86); NJ (T1 = 0.78, T2 = 0.87); NR (T1 = 0.82, T2 = 0.83). We did not assess facets at 3- and 6-month follow-up because they were included as mediators, and the mediation models could not be tested beyond post-treatment due to the waitlist control group commencing the intervention.

### Data Analyses

#### Sample Size Calculation

Lakens and Evers (2014) propose that in order to find a medium effect size between two groups, with power of 0.80, 41 participants are required in each group (Total *n* = 82). An a priori power analysis for an analysis of covariance (ANCOVA) computed using G*Power 3.1.9 (Faul et al. 2009) determined that a target sample size of 90 participants was required to sufficiently power the study at a 0.80 level to find a medium effect size.

#### Analytic Approach

Step 1: In service of our main study hypotheses (H1, H2, H3), we assessed the effect of the intervention on perceived stress, depression and anxiety immediately after intervention group participants had completed the course (end of the waitlist period for waitlist control participants). Step 2: To identify mindfulness facets to be included as mediators in subsequent analyses, we conducted a manipulation check to see if the intervention had affected one, some, or all of the facets. For steps 1 and 2, data were analysed using multivariate analysis of covariance (MANCOVA) and univariate ANCOVA in SPSS version 21 (IBM Corp 2012). Step 3: Using the PROCESS macro (Hayes 2013), we assessed the mindfulness facets affected by the intervention in a multiple mediation model. In our bootstrap analysis, we specified 10,000 resamples and 95% confidence intervals with confidence intervals including zero indicating a null effect (Mooney and Duval 1993). Step 4: Using repeated measures ANOVA, we assessed whether the effects of the intervention were maintained at 3-month and 6-month follow-up (H4). Step 5: We assessed clinically significant change using chi square tests for depression and anxiety because at baseline almost half of the sample were moderately to severely affected.

#### Multiple Imputation for Missing Data

Fifteen participants in the intervention group did not complete the intervention, and were recorded as drop-outs. All waitlist control participants completed the waitlist period. The dropout rate in the current study (25%; see Fig. 1) was comparable to other studies (for review, see Swift and Greenberg 2012). Best practice dictates that Intention-To-Treat (ITT) principles be adopted; whereby, all randomised participants are included in the analysis in their allocated groups, irrespective of treatment adherence or completion (Altman 2009). In the current study, missing data was imputed (five iterations) using the multiple imputation process in SPSS version 21 (IBM Corp 2012). In the multiple imputation model, in order to provide a good prediction of missing values, the following variables were entered: baseline (T1) scores for perceived stress, depression and anxiety; T1 scores for AA, DES, NJ, NR and demographic variables (age, gender, children [yes/no], time in current role [years], job status [full-time/part-time], job pattern [traditional/shifts], hours worked per week, level of education). We report ITT and Per Protocol (PP) results throughout in all Tables. We interpret our findings in respect of the ITT results, and only include reference to PP results in text where there is a significant difference.

#### Correlation Analysis

Prior to conducting the main analyses, correlation analysis was carried out on the main study variables and mindfulness variables in order to test the MANCOVA assumption that the main study variables would be correlated with each other—and that the mindfulness variables would be correlated with each other (Meyers et al. 2013). A meaningful pattern of correlations was observed amongst the main study variables, and also amongst the mindfulness variables (see Table 2).

**Table 2:** Correlations of all study variables at T1 and T2

		1	2	3	4	5	6	7	8	9	10	11	12	13	14
1	Stress_T1	–													
2	Stress_T2	0.45**	–												
3	Dep_T1	0.68**	0.39**	–											
4	Dep_T2	0.48**	0.82**	0.56**	–										
5	Anx_T1	0.73**	0.40**	0.79**	0.48**	–									
6	Anx_T2	0.48**	0.81**	0.49**	0.90**	0.52**	–								
7	Desc_T1	− 0.25**	0.02	− 0.24**	− 0.02	− 0.15	− 0.03	–							
8	Desc_T2	− 0.19*	− 0.18*	− 0.19*	− 0.25**	− 0.14	− 0.23*	0.59**	–						
9	AA_T1	− 0.31**	0.09	− 0.27**	0.05	− 0.36**	0.05	0.05	− 0.01	–					
10	AA_T2	− 0.16	− 0.42**	− 0.09	− 0.42**	− 0.17	− 0.44**	0.06	0.44**	0.06	–				
11	Non-J_T1	− 0.36**	0.15	− 0.26**	0.09	− 0.34**	0.09	0.17	0.04	0.63**	− 0.08	–			
12	Non-J_T2	− 0.37**	− 0.37**	− 0.33**	− 0.37**	− 0.33**	− 0.36**	0.28**	0.42**	0.08	0.38**	0.33**	–		
13	Non-R_T1	− 0.49**	− 0.37**	− 0.38**	− 0.36**	− 0.43**	− 0.43**	0.16	0.14	0.01	0.16	− 0.12	0.07	–	
14	Non-R_T2	− 0.46**	− 0.47**	− 0.36**	− 0.45**	− 0.39**	− 0.47**	0.27**	0.35**	0.04	0.37**	0.07	0.44**	0.49**	–

*Stress* perceived stress, *Dep* depression, *Anx* anxiety, *Desc* describing, *AA* acting with awareness, *Non-J* non-judging, *Non-R* non-reacting, *T1* baseline, *T2* post-treatment (Intervention Group participants) and post-waitlist period (Waitlist Control Group participants)

## Results

### Intervention Effects on Outcome Variables

In the MANCOVA analysis, T2 scores (end of course for the INT group, end of waitlist period for the WLC group) for perceived stress, depression and anxiety were entered as the dependent variables; T1 scores (baseline) were entered as covariates and group (INT, WLC) was entered as the predictor. Analysis showed a significant multivariate main effect for group, Wilks’ λ = 0.57, *F*(3, 111) = 28.34, *p* < 0.001, ƞ_p_^2^ = 0.43. ANCOVA analyses showed a significant reduction in perceived stress, *F*(1, 115) = 63.32, *p* < 0.001, *d* = − 1.25 (95% CI [− 1.64, − 0.85]); depression, *F*(1, 115) = 56.37, *p* < 0.001, *d* = −1.06 (95% CI [− 1.44, − 0.67]) and anxiety, *F*(1, 115) = 67.86, *p* < 0.001, *d* = −1.09 (95% CI [− 1.47, − 0.98]). Table 3 shows that the between-group effect sizes for all outcomes after course completion were large (Cohen 1988).

**Table 3:** Means, standard deviations (SDs) and between group effect sizes for Outcome Variables for Intervention and Waitlist Control Groups: Intention-to-Treat and Per-Protocol Analysis

	Perceived stress				Depression				Anxiety			
	INT group		WLC group		INT group		WLC group		INT group		WLC group	
	*n*	Mean (SD)	*n*	Mean (SD)	*n*	Mean (SD)	*n*	Mean (SD)	*n*	Mean (SD)	*n*	Mean (SD)
Intention to treat												
Before treatment (T1)	60	24.55 (5.53)	58	24.22 *(5.79)*	60	11.10 *(6.24)*	58	9.91 (5.93)	60	10.43 (4.96)	58	8.98 (5.32)
After treatment (T2)^a^	60	14.57 (5.45)	58	22.41 (7.00)	60	4.10 (4.10)	58	9.28 (5.57)	60	4.34 (3.94)	58	9.19 (4.93)
Effect size d [95% CI]^*b*^	− 1.25 [− 1.64, − 0.85]				− 1.06 [− 1.44, − 0.67]				− 1.09 [− 1.47, − 0.98]			
Per protocol												
Before treatment (T1)	60	24.55 (5.53)	58	24.22 (5.79)	60	11.10 (6.24)	58	9.91 (5.93)	60	10.43 (4.96)	58	8.98 (5.32)
After treatment (T2)^a^	45	15.02 (5.07)	58	22.53 (7.07)	45	4.52 (3.59)	58	9.31 (5.69)	45	4.63 (3.63)	58	9.18 (5.06)
Effect size d [95% CI]^*b*^	− 1.20 [− 1.61, − 0.77]				− 0.98 [− 1.38, − 0.56]				− 1.01 [− 1.42, − 0.59]			

*INT* intervention, *WLC* waitlist control, *T1* start of course (INT group) and start of waitlist period (WLC group)

^a^After treatment = end of mindfulness intervention (INT group) and end of waitlist period (WLC group)

^b^Between-group effect size represents pre- to post-intervention comparing the INT group at post-treatment to the WLC group at the end of the waitlist period

### Manipulation Check: Mindfulness Facets

In the MANCOVA analysis, T2 scores for describing, acting with awareness, non-judging and non-reacting were entered as the dependent variables; T1 scores were entered as covariates and group (INT, WLC) was entered as the predictor. Results showed a significant main effect for group, Wilks’ λ = 0.77, *F*[4, 109] = 7.97, *p* < 0.001, ƞ_p_^2^ = 0.23. ANCOVA analyses showed that the intervention significantly increased levels of AA, *F*(1, 115) = 42.94, *p* < 0.001, ƞ_p_^2^ = 0.27; DES, *F*(1, 115) = 5.76, *p* = 0.02, ƞ_p_^2^ = 0.05 (ITT) [*F*(1, 100) = 2.47, *p* = 0.12 (PP)] and NJ, *F*(1, 115) = 26.13, *p* < 0.001, ƞ_p_^2^ = 0.19. However, the intervention did not affect NR, *F*(1, 115) = 1.71, *p* = 0.19.

### Mediation Analysis

Separate multiple parallel mediation models were tested (see Table 4) whereby change scores for describing, acting with awareness and non-judging were entered simultaneously to assess whether they mediated the effect on the intervention on each of our outcome variables. For each outcome, in PROCESS, using model 4 (for multiple mediators): respective T2 scores were entered as the dependent variable (Y); Group (INT, WLC) was entered as the Independent variable (X) and describing, acting with awareness and non-judging were entered as mediators (M). In the top half of Table 4, a graphical representation of the mediation models for ITT and PP analysis are shown. The unstandardised Betas and standard errors (in brackets) for the effect of the intervention (intervention vs. waitlist control) on the mediators are embedded within this graphic on the relevant pathways. The bottom half of the table shows the effect of the mediators on each of the outcome variables. These figures correspond to the pathways in the graphic above from the mediators to the outcomes, with the A pathway being related to Describing, the B pathway being related to Acting with awareness and the C pathway relating to Non-judging. In order for a mediator to be significant, both pathways from the Intervention to the mediator from the mediator to the outcome must be significant.

**Table 4:**
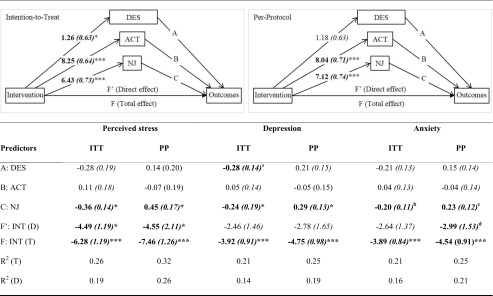
Intention-to-Treat and Per-Protocol Unstandardised Betas (Standard Errors) and explained variance (*R*^2^) for the indirect effects of the intervention on the outcomes (perceived stress, depression and anxiety) via the mindfulness facets (describing, acting with awareness and non-judging)

#### Perceived Stress

As can be seen in Table 4, only the *non-judging* facet of mindfulness operated as a partial mediator for the effect of the intervention. The direct pathway (F’, see Table 3) remained significant with the mediators in the model suggesting some remaining unexplained variance. The bias corrected bootstrap confidence interval for the indirect effect of *non-judging* on perceived stress (95% CI [− 4.33, − 0.61]) did not include zero; therefore, this was a significant effect.

#### Depression

The model explaining the effect of the intervention on depression was more complex. As can be seen in Table 4, both *non-judging* and *describing* appear to have operated as mediators for the effect of the intervention on depression, although the effect for *describing* was smaller than that for *non-judging*. Bias corrected bootstrap confidence intervals for the indirect effects of *non-judging* (95% CI [− 3.06, − 0.30]) and *describing* (95% CI [− 1.00, − 0.02]) on depression did not include zero; therefore, these were significant effects. Furthermore, the direct pathway was no longer significant with the mediators in the model suggesting this was a full mediation effect.

### Anxiety

Finally, with respect to anxiety, Table 4 shows that only the *non-judging* facet of mindfulness operated as a mediator. Bias corrected bootstrap confidence intervals for the indirect effect of *non-judging* on anxiety (95% CI [− 2.75, − 0.13]) did not include zero; therefore, this was a significant effect. As the direct pathway was no longer significant with the mediators in the model, this represented a full mediation effect.

### Analysis of Change over Time

We assessed the effect of the intervention over time for the outcome variables for the intervention group and for the waitlist control group separately. The waitlist control group completed the intervention after the waitlist period and were also followed-up 3 and 6 months after they completed the intervention. We were interested to see if waitlist control participants reported improvements that were similar to those reported by intervention participants. Table 5 shows that, for both groups, there was a significant main effect of the mindfulness intervention over time for all of the outcome variables (with large effect sizes; Cohen 1988). Furthermore, for all outcomes—when compared to baseline scores—the differences at post-treatment, 3- and 6-month follow-up were significant and were also associated with large effect sizes (Cohen 1988).

**Table 5:** Intention-to-treat and per-protocol repeated measures ANOVA results and within group effect sizes for outcome variables for intervention and waitlist control groups

	Intention to treat analysis				Per protocol analysis			
	INT group		WLC group		INT group		WLC group	
	*F*	ƞ_p_^2^	*F*	ƞ_p_^2^	*F*	ƞ_p_^2^	*F*	ƞ_p_^2^
Perceived stress (main effect)	48.67*	0.45	57.00*	0.50	35.24*	0.46	40.88*	0.51
T1 vs. T2	158.56*	0.72	140.17*	0.71	112.09*	0.73	94.93*	0.70
T1 vs. T3	46.35*	0.44	63.34*	0.54	35.49*	0.46	45.28*	0.53
T1 vs. T4	50.13*	0.45	80.42*	0.58	33.27*	0.44	52.87*	0.57
Depression (main effect)	32.31*	0.35	41.94*	0.42	21.94*	0.34	31.91*	0.45
T1 vs. T2	80.25*	0.58	67.05*	0.54	52.24*	0.55	61.65*	0.61
T1 vs. T3	20.58*	0.27	50.77*	0.47	13.25*	0.24	41.83*	0.52
T1 vs. T4	48.97*	0.45	58.77*	0.51	34.89*	0.45	39.45*	0.50
Anxiety (main effect)	38.55*	0.39	39.16*	0.41	29.49*	0.41	27.87*	0.42
T1 vs. T2	111.53*	0.65	59.35*	0.51	76.56*	0.65	49.19*	0.59
T1 vs. T3	35.65*	0.38	48.58*	0.46	32.78*	0.44	34.69*	0.47
T1 vs. T4	64.13*	0.52	59.56*	0.51	50.19*	0.54	41.09*	0.51

*INT* intervention, *WLC* waitlist control, *T1* before mindfulness course, *T2* after mindfulness course, *T3* 3 month follow-up, *T4* 6 month follow-up

*p < 0.001

### Clinically Significant Change

Chi square tests showed a significantly smaller proportion of participants allocated to the INT group, as compared with those allocated to the WLC group, continued to report moderate/severe levels of depression and anxiety immediately after the intervention was completed (see Table 6). Based on the odds ratio, the odds of participants in the WLC group reporting moderate/severe depression and anxiety were six to seven times higher than participants in the INT group immediately after the mindfulness intervention had been completed. For perceived stress, we assessed the post-treatment scores for each group against the normative mean for the PSS-10 (*X* = 13.02; Cohen et al. 1983; Cohen and Williamson 1988), and found that while both groups reported perceived stress levels which were significantly higher than the normative mean (WLC: *X* = 22.41, *t*[57] = 10.204, *p* < 0.001; INT: *X* = 14.57, *t*[59] = 2.197, *p* = 0.03), the mean PSS score in the intervention group was approaching the normative value at the end of the intervention.

**Table 6:** Intention-to-Treat and Per-Protocol Analyses of the Proportion of Participants in the Intervention and Waitlist Control Groups meeting criteria for moderate to severe levels of anxiety and depression at post-intervention

	INT group		WLC group			
	*n*	%	*n*	%	*X* ^2^	OR
Intention-to-treat						
Moderate/severe depression	6	10	26	44.8	18.09*	7.89
Moderate/severe anxiety	7	11.7	27	46.6	17.50*	6.64
Per-protocol						
Moderate/severe depression	4	8.7	25	45.5	16.54*	8.75
Moderate/severe anxiety	5	10.9	25	45.5	14.35*	6.83

*OR* odds ratio, *WLC* waitlist control, *INT* intervention

*p < 0.001

### Impact of Course Completion Time

Participants were encouraged to complete the course within 4 weeks of their start date; however, there was variation with regards to time taken to complete the course. The average time participants in the intervention group took was 6 weeks and 3 days, and all had completed the course within 12 weeks. In detail, 11.1% (*n* = 5) completed within 4 weeks, 62.2% (*n* = 28) completed within 6 weeks, 84.4% (*n* = 38) completed within 8 weeks, 95.5% (*n* = 43) completed within 10 weeks and 100% (*n* = 45) completed within 12 weeks. In order to assess whether there were differences in the effect of the intervention due to time taken to complete the course, data from participants in the intervention group was split into those who completed within 6 weeks (*n* = 30), and those who took longer than 6 weeks to complete (*n* = 15). A series of *t* tests were performed and results showed no significant differences between those who completed the course within 6 weeks and those that took longer than 6 weeks for any of the study outcomes.

## Discussion

Results showed that participants who completed the online mindfulness course reported significantly lower levels of perceived stress, depression and anxiety. The large effect sizes associated with completing the intervention were maintained for all of the outcome variables at 3- and 6-month follow-up. The effect sizes in this study rival those of studies which have employed a group-based face-to-face format for mindfulness-based intervention delivery (e.g. see Nyklicek and Kuijpers 2008), and they align with the findings from Krusche et al. (2012) in their pre-post study design. However it is important to note that the relatively high baseline levels reported in this sample meant there was more capacity for change from baseline, and our results are contrary to the small (depression, anxiety) and medium (stress) effect sizes reported in the recent meta-analysis by Spijkerman et al. (2016). Nonetheless, it is possible these differences are a reflection of the different online mindfulness interventions being assessed. For example, all of the studies included in Spijkerman et al.’s meta-analysis were MBSR or Acceptance and Commitment Therapy (ACT), whereas the intervention assessed in this study was an MBCT intervention. Perhaps MBCT has proven more effective when operationalised online than the other mindfulness-based interventions. However, this is speculative and further empirical work is needed. In addition, Spijkerman et al. included studies with student, general population and clinical samples; therefore, the reported effect sizes for depression, anxiety and stress may have been influenced by differences conferred by the different sample types.

A number of authors have called for research designed to understand by what mechanism/s mindfulness exerts its positive influence (e.g. Brown et al. 2007; Glomb et al. 2011). Our results showed that the online mindfulness course exerted its effect on the outcome variables predominantly through increased levels of one facet of mindfulness; that is, increased levels of *non-judging* (although *describing* did contribute to the model for depression). While the intervention worked to increase levels of other facets of mindfulness (*acting with awareness and describing*), these facets did not mediate the change in the outcome variables, and *non-reacting* did not appear to be affected by the intervention. These findings are of interest for a number of reasons. It is curious that the mindfulness intervention did not affect all of the mindfulness facets, and two other studies have shown similar findings. From the occupational health literature, Querstret et al. (2016) found that three out of the four mindfulness facets were affected by the intervention (acting with awareness, describing, and non-judging); however, only one facet (acting with awareness) mediated the change in work-related rumination, fatigue and sleep quality. From the clinical literature, Boden et al. (2012) found that the impact of their intervention on post-treatment posttraumatic stress disorder severity was mediated by *acting with awareness*; whereas, the impact on post-treatment depression severity was mediated by *non-judging*. These differing findings raise an interesting possibility that the different facets of mindfulness are more or less important with regards to their impact on different health outcomes.

If only some of the mindfulness facets are implicated in mediation models perhaps interventions targeting those facets would be useful. However, this is a cautious proposal as more research is needed to understand how the different facets relate to one another. For example, Querstret et al. (2016) posited that some facets may develop earlier in mindfulness training (e.g. observing, describing and acting with awareness), with the remaining facets developing when participants are more skilled (e.g. non-judging, non-reacting). If this is the case, it might be that study designs are not long enough to capture the change in non-reacting (for example) because it may develop though continued practice after the study has ended.

Our study was conducted in a general population sample; however, in both study groups approximately half the sample reported moderate to severe depression and anxiety at baseline. All participants were working at the time of taking part in the study suggesting a relatively high level of functioning. In the context of research suggesting that much of the burden of disability in the population is attributable to subclinical symptoms (Judd et al. 2002); intervening early before depression and anxiety increase to clinically diagnosable levels could be beneficial for the individual (i.e. by keeping them in work and feeling productive and healthy) and to health services, by reducing the number of people needing to engage with more complex psychological therapy and other forms of intervention (e.g. drug therapy). Therefore, operationalising therapies (like mindfulness) online could increase their availability, reduce waitlist times and reduce cost to health services.

### Limitations

An inherent limitation in waitlist control designs is that they do not allow for multiple treatments to be assessed against each other; therefore, the effects in this study may reflect a general treatment effect. However, the effect sizes in this study are comparable to those in studies considering mindfulness in randomised controlled trails (e.g. van Aalderen et al. 2012; Vollestad et al. 2011). Data concerning the amount of meditative practice participants engaged in over the course of the study was not collected which makes it difficult to assess whether the amount of practice participants engaged in was a mechanism of change. For example, the moderate to large effect sizes found in the current study may be an artefact of a very motivated cohort, practicing consistently many hours and days a week.

The moderate to severe levels of self-reported depression and anxiety in both groups at baseline does raise questions about the generalisability of our findings to other general population samples. We did not seek to recruit a clinical sample; however, many of our participants were recruited from industry sectors which may be inherently stressful to work in (e.g. healthcare, education, financial services, information technology, telecommunications). It is also more likely that individuals experiencing higher levels of distress would be more attracted to an intervention for health and wellbeing, so the baseline levels may also reflect a tendency for individuals who need intervention to self-select into these types of studies. The compensation offered to participants for taking part in the study (£50 worth of shopping vouchers) was not insubstantial and may have kept participants engaged in the study, masking the true dropout rate. It may also have influenced the generalisability of the sample with motivation for taking part being linked to the reimbursement.

Related to issue of large effect sizes, the current study has demonstrated some significant mediation effects with a relatively modest sample size (*n* = 118). However, the failure to detect more modest mediation effects may have been the result of relatively low power for such complex mediational analyses rather than the effects themselves not existing (see Fritz and MacKinnon 2007). This is clearly an empirical question for future research so it is premature to conclude that only non-judging is the active ingredient in mindfulness interventions of this sort. Finally, we need to exercise caution in claiming causal mediating relationships since assessment of change was based on changes in variables measured at the same time points. We cannot entirely rule out the possibility that changes in our outcome variables caused the changes in our putative mediators.

### Future Research

Given the findings in this study showing the only one facet of mindfulness (*non-judging*) predominantly accounted for the effects of the intervention on the outcome variables, it would be useful to replicate this study in different samples to assess the stability of these findings. It would also be useful to conduct other studies with varying outcome variables, from different health domains, to further understand if the different mindfulness facets are specifically related to different conditions or health domains. This could then enable the development of interventions that are also condition/domain specific. Ideally, future mediation studies should attempt to show that changes in the mediators occur temporally prior to changes in the outcomes. For example, Kazdin (2007) recommends that both mediators and outcomes are measured several points throughout treatment to establish whether the mediator changes prior to any change in the outcome variable(s). Furthermore, because the results showed no difference between participants who took less than 6 weeks to complete the course and those who took longer than 6 weeks, developing shorter interventions may be fruitful. Further empirical work is needed with larger RCTs and well-chosen active and inactive control groups in order to understand the generalisability, implementation challenges, and cost-effectiveness of the intervention assessed in our study. Further empirical work assessing online mindfulness interventions against face-to-face group-based mindfulness interventions is also warranted to understand the relative contribution of social support offered in group-based formats.
